# Efficacy of Hyaluronic Acid in The Selection of Human Spermatozoa with Intact DNA by The Swim-up Method 

**DOI:** 10.22074/cellj.2016.3990

**Published:** 2016-04-04

**Authors:** Aslihan Saylan, Selcuk Duman

**Affiliations:** Necmettin Erbakan (NE) University, Meram Faculty of Medicine, Department of Histology and Embryology, Konya, Turkey

**Keywords:** Annexin V, Apoptosis, Hyaluronic Acid, Human

## Abstract

**Objective:**

In 2014, enrolled 20 patients who applied to the Unit of Assisted Reproduction Techniques, Konya Necmettin Erbakan University. Based on the presence of hyaluronic acid (HA) in the oocyte-cumulus cell complex, sperm attached to HA *in vivo* were modeled *in vitro*. Available healthy sperm obtained in the swim-up procedure using HA were investigated.

**Materials and Methods:**

This observational cohort study, a routine analysis was conducted on the ejaculation samples obtained from 20 patients. We divided each sample into two groups and the swim-up method was applied. Human serum albumin (HSA, 0.5%) was added to samples from the first group. HA (10%) was added to samples from the second group. We determined the floating linear and non-linear sperm concentrations of both groups annexin V was used to determine the rate of apoptosis of these sperm.

**Results:**

Following swim-up, linear and non-linear sperm concentrations were higher in the group that contained HA compared to the group with HSA. However, there was a significantly higher apoptosis rate in the HSA group compared to the HA group.

**Conclusion:**

The addition of HA to the medium in the swim-up procedure positively affected sperm parameters. Thus, healthier sperm cells were obtained without DNA damage and with high motility.

## Introduction

Infertility is a problem that affects approximately 8% of reproductive age men (1). Semen analysis performed to evaluate infertile men (volume, pH, concentration, motility, morphology, and number of leukocytes) is a basic laboratory evaluation (2), which has been used in the standard analysis of male infertility; however, these parameters are inadequate in the definitive differentiation between fertile and infertile men (3). Sperm aneuploidism and DNA fragmentations are seen at higher rates in infertile men compared to fertile men (4). High rates of DNA damage are frequently associated with poor semen parameters and infertility; however, sperm DNA damage can also occur in men with normal sperm analysis (5). Abnormalities of sperm morphology and motility are to be associated with DNA damage (6,7), negatively affect fertilization of sperm cells with damaged DNA (8,9), impair embryo quality, and increase the rate of abortions (10). Various studies have demonstrated abnormalities of sperm morphology and DNA damage (11,12). 

There are many methods to select sperm cells, including: i. Apoptosis (magnetic cell sorting and glass wool), ii. Surface charge (electrophoresis and zeta potential), iii. Membrane maturity [hyaluronic acid (HA) binding], and iv. Ultramorphology (high magnification). Better quality sperm selection is possible by the application of the previously mentioned methods (13). 

One of the traditional techniques in preparing human spermatozoa is the swim-up method. The advantages of the swim-up method are its similarity with *in vivo* sperm selection and its low decoded rates which have the advantage of taking less time (14). 

HA is a linear anionic polysaccharide composed of N-acetyl-D-glucosamine and D-glucuronic acid bound with β-1-3 and β-1-4 glycosidic bonds (15). 

HA is related to the glycosaminoglycan family. It is a natural macromolecule, present in high amounts in human body fluid and the extracellular matrix (16). HA, which is one of the main compounds of the cumulus cell matrix that surrounds the human oocyte (17), holds cumulus cells together by forming a labyrinth system in the process of the mature sperm that reaches the oocyte during fertilization (18). Mechanisms associated with the role of HA for selection of sperm with high fertilization capacity in the natural fertilization process have been proven by research. Therefore, HA has been accepted as a sperm selector in physiological intracytoplasmic sperm injection (ICSI) applications (19). HA, when added to the sperm preparation media, increases sperm motility and preserves sperm motility of fresh semen and frozen/defrosted human sperm during the incubation period (20). 

Therefore, HA can be used in the swim-up medium in place of the routinely used human serum albumin (HSA) (21). The addition of HA to swim-up medium is a simple, inexpensive technique (22). 

Based on the hypothesis of the inversely proportional number of sperm that penetrate into HA and apoptosis, we have aimed to obtain healthier sperm by the addition of HA to the swim-up medium. 

## Materials and Methods

This observational cohort study enrolled normospermic individuals, according to the World Health Organization (WHO) 2010 criteria, who were among patients that had applied to the Unit of Assisted Reproduction Techniques at Konya Necmettin Erbakan University, Meram Faculty of Medicine. A total of 20 normospermic patients were chosen based on the Ethics Committee of Meram Medical Faculty decision number 2014/645. 

### Preparation of semen samples

Semen samples obtained from the patients after three days of sexual abstinence were used according to the WHO 2010 Criteria. Samples were collected in sterile, non-toxic polypropylene containers following a 25-minutes duration of liquefaction. First, a physical examination was performed to evaluate the smell, color, volume, and viscosity of the semen. Subsequently, sperm concentration, motility, and morphology were evaluated using a Makler counting camera (Sefi Medical Instruments) under a Nikon T1A Input AC light microscope. 

### Sperm obtained through routine swim-up method with hyaluronic acid

Semen samples included in this experiment were analyzed without delay. We used Hank’s Balanced Solution (HBSS, Biological Industries, catalog no: 02-017-1) with bicarbonate tamponed as the basic flushing fluid. The liquefied semen sample of the patient was mixed with HBSS in a 1:1 proportion following routine semen analysis. Each sample was divided into two separate sterile, conical 14 ml tubes (Nest, catalog no: 601002). The HBSS+semen samples were centrifuged at 1000 rpm for 10 minutes. Following centrifugation, the supernatant was removed, the pellet was homogenized and subsequently divided into 1 ml Eppendorf tubes with 250 µl of sample in each tube. For the first tube, we added HBSS (used in the routine swim-up method) that contained 0.5% HSA and for the second tube HBSS with 10% HA was added using a method of leakage to the wall of the tube, forming two separate layers. 

Finally, Eppendorf tubes were placed in an incubator with 5% CO_2_and were left there to allow the sperm cells to swim to the top layer. After 45 minutes, samples from the sperm cells that had swam up to the swimming fluid which contained HA and HSA were counted by a Makler camera for linear progressive (+4) and non-linear (+3) motility. The entire experiment was performed at room temperature. 

### Evaluation of sperm apoptosis (annexin V staining)

Phosphatidylserine translocates from the inner layer to the outer layer of the sperm membrane during the early stages of apoptosis. The annexin V antibody is a Ca^+2^dependent phospholipid-binding protein that binds specifically to the externalized phosphatidylserine molecule. Fluorescein created by the phosphatidylserine that binds to annexin V can be used as a marker for sperm apoptosis (23). 

In order to evaluate sperm apoptosis, samples obtained in each group (HSA, HA) from the swim up technique were transferred into 1 ml Eppendorf tubes such that each tube would contain one million total cells. Subsequently, cells were treated with an annexin-V-Fluos Staining Kit (F. HoffmannLa Roche, catalog number: 11858777001), homogenized, and allowed to remain for 30 minutes. 

Approximately 45-50 μl of the mixture was dropped on each slide (poly L-lysine). A lamella was placed on top of each lam after which, the mixture was evaluated in the dark under a fluorescent microscope with an Olympus BH-2 photo attachment. A total of 100 cells were counted. From those, we considered the red cells to be necrotic, red+green cells were late apoptotic cells, and green cells were evaluated as early apoptotic cells. The cell group that showed no stain color was considered to be the healthy cells group. The entire experiment was performed at room temperature. Statistical analyses were performed after all data were obtained. 

### Statistical analysis

The statistical analysis of the study was performed using an IBM SPSS (Version 22.0. Armonk, NY: IBM Corp.). We used the one-way ANOVA test to observe the differences between the colors obtained as a result of the annexin V challenge. The numbers of floating sperm cells following HSA and HA treatment were analyzed to evaluate motility. To observe the differences in the mean values of HA and HSA, we conducted the paired sample t test. Data were evaluated at P<0.05 a confidence interval and analyzed. 

## Results

This study was performed on 20 patients who applied to the Unit of Assisted Reproduction Techniques of the Konya Necmettin Erbakan University, Meram Faculty of Medicine for semen analysis. Among those individuals who applied for semen analysis, the patients who were normospermic, according to the WHO 2010 criteria and designated during routine semen analysis were included in this study. 

## Evaluation of sperm concentration

There were statistically more +4 and +3 sperm cells in medium that contained 10% HA compared to +4 and +3 sperm cells in medium that contained 0.5% HSA (P<0.05). 

## Morphological evaluation of sperm

We conducted morphological evaluation of the sperm in swim-up medium by treating the samples with annexin V and subsequently evaluating them under a fluorescent microscope. Sperm cells with +4 and +3 motility in the medium that contained HSA were morphologically compared with the cells that had +4 and +3 motility in the medium with HA. The fresh form of the semen was added to the comparisons. The necrotic cell ratio was found to be statistically higher in the group that contained HSA compared to the group with HA (P<0.05). A comparison between the two groups showed a statistically higher ratio of late apoptotic cells in the HSA group (P<0.05). However, when the ratio of early apoptotic cells in these two groups were compared, no statistically significant difference was found. When the fresh semen sample was compared with sperm cells that contained HSA in terms of ratios of apoptotic and necrotic cells, there was no significant difference, whereas a statistically significant difference was found when the fresh semen sample was compared with the group that contained HA (Table 1). 

**Table 1:** Percentages of sperm concentrations with +3 and +4 motility among the sperm cells swimming in Hank’s Balanced Medium that contained HSA or HA


Non-stained	Green	Red+green	Red

Non-apoptoticcell	Early phase apoptotic cell	Late phase apoptotic cell	Necrotic cell


HSA; Human Serum Albumin and HA; Hyaluronic Acid.

We compared the ratio of healthy sperm cells without staining between the groups following annexin V treatment. There was a statistically significant ratio of healthy sperm cells in the HA group compared to the fresh semen sample and the HSA group (P<0.05, Figes.1-3). 

**Fig.1:**
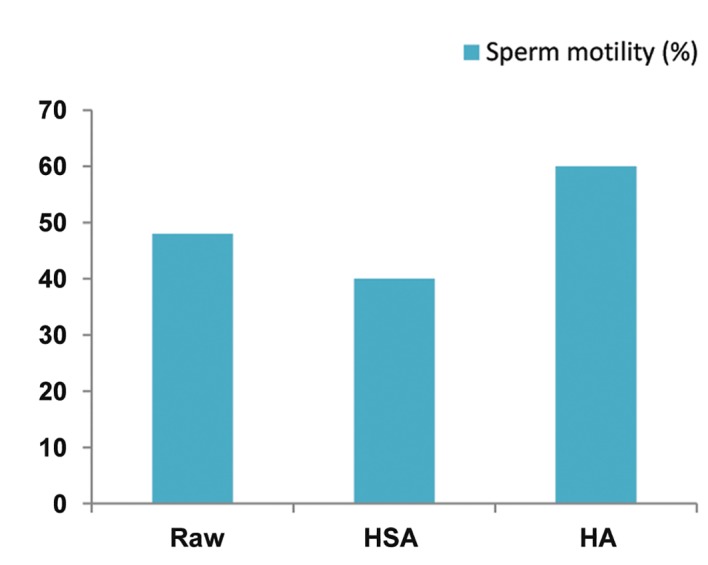
Cell interpretation under fluorescent microscope. Concentrations of sperm cells in medium that contained 10% hyaluronic acid (HA) were significantly higher compared to the concentration of sperm cells in medium that contained 0.5% human serum albumin (HSA, P<0.05).

**Fig.2:**
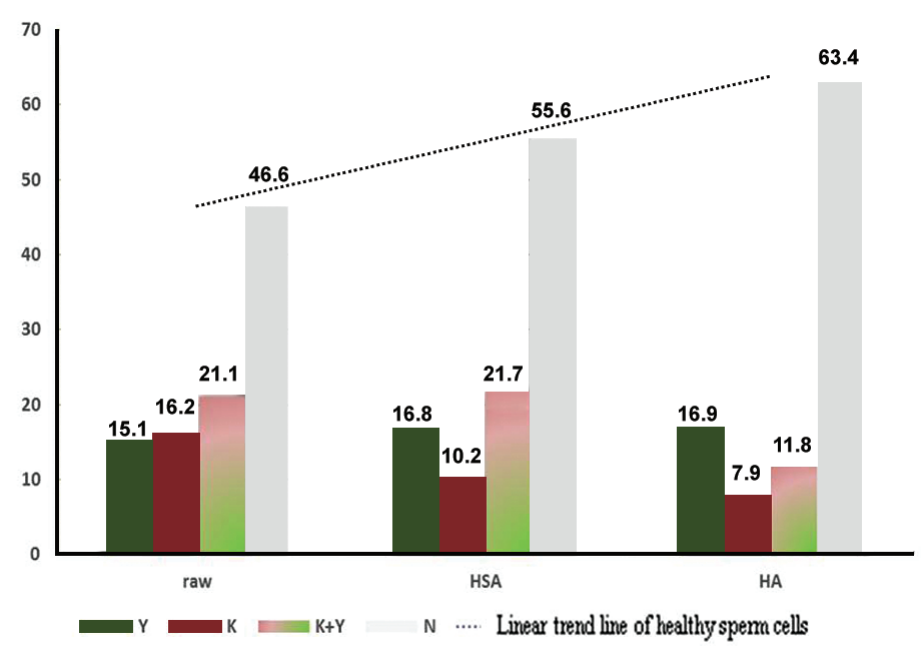
Percentage of morphologic states of fresh semen, linear progressive and non-linear sperm cells obtained following staining with annexin V after treatment with human serum albumin (HSA) and hyaluronic acid (HA). Y; Green, K; Red and N; Normal.

**Fig.3:**
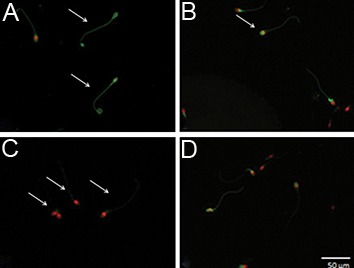
A. Swim-up method. Floating human sperm cells were stained with annexin V. Early apoptotic cells (green) in swimming fluid that contained 10% hyaluronic acid (HA) , B. Late apoptotic cells (red+green) in swimming fluid that contained 0.5% human serum albumin (HSA), C. Necrotic cells (red) in swimming fluid containing 0.5% HSA and D. Spermatozoa samples that were annexin-V stained (scale bar: 50 μm).

## Discussion

Fertilization begins with the merging of female and male gametes through the fusion of cell membranes that result from the interaction of the sperm and egg. The completion of this process and subsequent embryo development are dependent on sperm DNA integrity (14). There is a risk of fertilization of the oocyte by a sperm with a damaged DNA. Therefore, sperm DNA gains importance under conditions such as implantation failure or abortion during the early periods, despite successful fertilization (24). DNA integrity and sperm morphology have been found to be more reliable compared to other sperm parameters (25). Caspase activation, phosphatidylserine externalization, mitochondrial membrane potential change, and DNA fragmentation in ejaculated sperm have been accepted as markers of apoptosis. Studies indicate that infertile men have a higher rate of apoptotic sperm compared to fertile men. DNA damage to the sperm is an important predictor of potential of fertility (26,28). Binding of the sperm to HA demonstrates the developed maturity of the sperm, scarcity of chromosomal damage, and high rate of DNA integrity of that sperm. It predicts the fertilization ability of the sperm (22). In the current study, we have added HA to the swim-up medium which allowed us to obtain healthier sperm compared to HSA, which has been used in routine applications. The progressive spermatozoa concentration swimming to the HA-supplemented medium yielded a higher number of healthy sperm. In addition, those spermatozoa were proven to be healthy spermatozoa with intact DNA which had a low rate of apoptosis after treatment with annexin V, a marker of apoptosis. In a similar study, Jakab et al. (29) reported that they obtained healthier sperm and a decreased rate of chromosomal abnormalities during an ICSI procedure that used HA. Similarly, Park et al. (30) compared the ICSI results of tests conducted using selected sperm bound to HA. They indicated that the rate of chromosomal abnormalities was lower in the embryos obtained with selected sperm bound to HA. This finding positively affected the pregnancy results. In a study performed in oligospermic patients, Parmegiani et al. (19) found a higher rate of nuclear abnormalities in the group of sperm bound to HA compared to the group not bound to HA. Petersen et al. (18) observed that HA was inadequate for sperm selection with normal morphology under large magnification. Huszar et al. (31) proved that sperm bound to HA have developed sperm maturity and high sperm integrity in addition to a full acrosome, mature nuclei, and good morphology. This simultaneously has demonstrated that sperm bound to HA are mature and have completed the plasma membrane renewal, spermatogenetic processes, and nuclear histoneprotamine exchange. Ye et al. (32) evaluated the association between the rate of HA bonding and fertilization. They reported a strong correlation between the HA binding rate with total and progressive sperm motility and morphology. Tarozzi et al. (33), on the other hand, evaluated hyaluronanbound (HB) sperm values and rate of DNA fragmentation in 60 *in vitro* fertilization (IVF) sperm in order to analyze the use of HB in male fertility. They found no correlation between HB values and fertilization-implantation rates, however a high correlation between HB values and sperm morphology was observed (P<0.05). They observed a low rate of DNA fragmentation in the group with high HB values (P<0.05). 

## Conclusion

Sperm obtained from the HA supplemented medium have more motility, a higher rate of DNA integrity, and better morphologic quality. The chance of a sperm with these specifications penetrating the oocyte is higher. We suggest that healthier embryos can be obtained with the use of sperm with intact DNA that are obtained by this method. This simple, cheap, and easily performed method which has the potential to be routinely used can be performed using basic media with HA support and may contribute to the assisted reproductive technology (ART)-andrology. 
